# The diagnostic role of cardiovascular magnetic resonance imaging in patients surviving non traumatic out of hospital cardiac arrest

**DOI:** 10.1186/1532-429X-16-S1-P220

**Published:** 2014-01-16

**Authors:** Nauman Ahmed, Amardeep G Dastidar, Elisa McAlindon, Chris Lawton, Mark Hamilton, Nathan Manghat, Julian Strange, Chiara Bucciarelli-Ducci

**Affiliations:** 1 Cardiology, Bristol Heart Institute, Bristol NIHR Cardiovascular Biomedical Research Unit (BRU), Bristol, UK

## Background

Out of hospital cardiac arrest (OOHA) is one of the leading causes of death in western world. Acute coronary syndrome (ACS) is the most common aetiology of OOHA but at least one third of OOHA have unobstructed coronaries on angiography. Cardiovascular magnetic resonance (CMR) non invasive tissue characterisation has the potential to establish the differential diagnosis in patients with OOHA.

## Methods

We retrospectively reviewed the database in our tertiary cardiothoracic centre from October 2009 to September 2013. We identified 50 consecutive patients who were referred for a CMR following an OOHA. A comprehensive CMR protocol was used (including T2 weighted STIR imaging for oedema, adenosine perfusion and late gadolinium enhancement imaging for myocardial scarring). All scans were done within 6-8 weeks of the index event and was reported by a consultant with >10 years of experience in CMR.

## Results

Out of the 50 patients (14 female, 36 male, age range 21-80 years, n = 26 (52%) had coronary artery disease in which the culprit was treated by primary angioplasty and n = 24 patients had unobstructed coronaries. Of these n = 3 had hypertrophic cardiomyopathy, n = 3 had myocarditis/cardiac sarcoid, n = 2 had non-ischaemic dilated cardiomyopathy, n = 2 had LV non compaction (LVNC) cardiomyopathy, n = 7 had nonspecific abnormalities and n = 9 had completely normal CMR scan. In 68% of cases, a cause for the OOHA was found. In all patients (n = 26) with significant CAD on angiography CMR identified a myocardial infarction (100%). In patients with bystander CAD (n = 17), n = 13 (76%) had either inducible perfusion defect or viability addressing the patients to a staged revascularization approach

## Conclusions

In adults surviving a non-traumatic OOHA CMR outlined the underlying condition (ischemic and non-ischemic cardiomyopathy) in the large majority of cases. Establishing a final diagnosis has implications for treatment and prognosis.

## Funding

None.

